# Optimal management of oil content variability in olive mill batches by NIR spectroscopy

**DOI:** 10.1038/s41598-019-50342-6

**Published:** 2019-09-27

**Authors:** E. C. Correa, J. M. Roger, L. Lleó, N. Hernández-Sánchez, P. Barreiro, B. Diezma

**Affiliations:** 1 Laboratorio de Propiedades Físicas y Técnicas Avanzadas en Agroalimentación (LPF_TAGRALIA). Escuela Técnica Superior de Ingeniería Agronómica, Alimentaria y de Biosistemas, Universidad Politécnica de Madrid, CEI Moncloa. Avda. Puerta de Hierro 2-4, 28040 Madrid, Spain; 2 Chemhouse Research group, ITAP, Univ Montpellier, Irstea, Montpellier SupAgro, Montpellier, France

**Keywords:** Near-infrared spectroscopy, Infrared spectroscopy

## Abstract

Total oil content (OC) is one of the main parameters used to characterize the whole of olives entering a commercial mill, quantified by the total fresh weight of the lot and the oil concentration (%) assessed in a representative sample on olive paste, by means of chemical extraction. Nuclear magnetic resonance (NMR) and NIR spectroscopy are alternative methods even at individual olives. This work evaluates several strategies to calibrate precise NIR models for the estimation of the total OC. To this end, 278 olives were analysed covering whole season variability in terms of olive fresh-weight and the corresponding OC by chemical extraction in 31 batches. The average spectra from hyperspectral NIR images (1003–2208 nm) were computed for each fruit and the actual OC (g) of those olives determined by NMR (0.09 to 1.29 g with a precision of 0.017 g). According to the results, current batch based assessment of the OC (Soxhlet, %) in mills only reproduces 44% of the underlying heterogeneity, despite being the factory standard. The incorporation of individual NIR spectra (278) to the 31 Soxhlet values of the batches allows a 67% explanation of the OC (%) of olives. When estimating OC (g) gathering individual fresh weight and the estimation of oil concentration in olives, a standard error of prediction of 0.061 g is reached (r^2^ = 0.93), a precision value that approaches the potential limit according to the NMR reference (0.017 g).

## Introduction

Oil quality and content in olives depend on complex agricultural factors that determine the ripening process of fruits^1^. On the other hand, the oil content (OC) determines the adjustment of key parameters of the milling process^2^. The estimation of the average oil content of incoming olive batches in a mill constitutes the basis of oil extraction control in terms of duration and temperature of malaxation^3^, and of the rate of feeding in the decanter pump^4^. All of which ensure the optimization of oil extraction: improvement of yield (litres per kg) and quality of olive oil^4^.

Currently, gravimetric analysis under Soxhlet extraction is the official method to determine the OC in olive batches^5^, however, it is time consuming, and requires sample preparation and dissolvents. More recently, nuclear magnetic resonance (NMR) is being used in quality laboratories to determine OC. Both methods make use of olive paste in such environments.

García, *et al*.^6^ have shown that either NMR, or Soxhlet extraction provide comparable estimations in oil concentration for milled olives, and thus both methods are taken for redundant.

NIR spectroscopy is an alternative method for oil content quantification increasingly used in quality laboratories, and industries for routine analysis (commercial equipment Foss OliviaTM; Bruker MPA). It is fast and does not require dried samples, but it is most frequently applied on olive paste^6,7^. More recently, innovative implementations of NIR systems for OC quantification have been conducted on intact olives, for breeding programs^8,9^ where the selection of individuals becomes the main target, or for the evaluation of fruit entering the mill^10–12^. In addition, advances in NIRS technology have allowed the evolution from laboratory equipment^13,14^ to the implementation of in-field portable devices^15^ as well to on-line spectrometers^16^, leading to faster and more efficient analysis compared to laboratory NIRs. Still, NIR remains an indirect method that requires a rigorous calibration procedure to be implemented.

Moving from mill batches, to small samples or to individual olives requires the consideration of concepts such as homogeneity/heterogeneity from the point of view of the theory of sampling^17^. Different sampling strategies require different management of heterogeneity.

In some studies, batches constitute the decision units with two alternative procedures: using homogenized samples such as pastes (composite samples), or the use of intact fruits in the case of non-destructive methods^11,12,16^.

NMR and chemical extraction protocols (factory standard)^13,18,19^ have also been implemented to determine the oil content of individual olive fruit. Comparisons of the official methods with regard to NMR quantification of the oil content in olives has demonstrated that NMR presents the highest overall efficiency (more sensitivity, good repeatability and higher precision)^14,18^. Thus, NMR is a more direct procedure for the calibration of NIR models.

At the industrial level, computer vision is a widely used technology in the production of table olives to determine the fruit size and detect external damage (H2020-SMEInst-2018-2020-2 Project: Evoolution). The possibility of online scanning of all the olives to be processed in a mill, either by multi or hyperspectral image systems (VIS and NIR)^20^, opens the doors to have a very accurate and real-time information of the flow of the oil that effectively enters the industrial process, even allowing previous segregation of individual fruits in more homogeneous batches.

The purpose of this work was to evaluate different strategies to calibrate NIR models in practical situations in which the decision units are individual olive fruits for NIR, and olive paste for the reference method. This paper addresses, as an innovation compared to previous literature, three approaches managing different levels of heterogeneity in the references considered to estimate oil concentration (%) and total oil content (g).

## Materials and Methods

### Sampling and methods

Olive fruits from a commercial mill in Toledo (D.O. Montes de Toledo), belonging to the varieties Arbequina, Picual and Cornicabra, were harvested with a wide maturity range at 12 harvest dates from November 2015 to February 2016. In total, 278 olives were clustered in 31 batches according to the harvest date, variety and maturity level (regarding to the external colour of the olives: green, purple and black).

**Figure 1:**
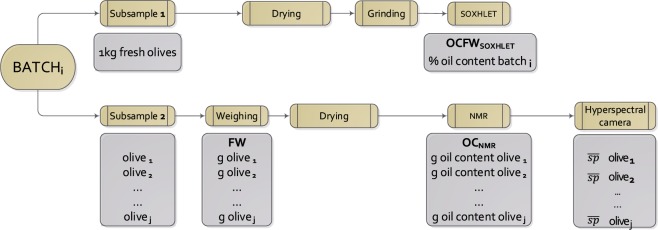
Scheme of the sampling and methods implemented. FW: fresh Weight (g); OCFW: oil content fresh weight (%); OC: oil content (g); $\overline{sp}$: average spectrum per fruit.

Once the olives were harvested and classified into 31 batches of similar maturity, they were immediately moved to the LPF_TAGRALIA laboratory in Madrid (Spain). Each batch or sample unit was divided into two subsamples (Fig. 1): Subsample 1 (1 kilogram of fresh olives) was sent to the reference laboratory CM Europa S.L. (Jaén, Spain) to undergo reference analysis of the oil content on a fresh-weight basis using Soxhlet (OCFW_SOXHLET_ (%)); Subsample 2, constituted by 8–9 individual olives, was used for NIR analysis.

In LPF_TAGRALIA facilities, the fresh weight (FW(g)) of each fruit from Subsample 2 was measured using a precision balance (ADP 720/L; Adam Equipment Co. Ltd., Kingston, Milton Keynes, UK). Next, the fruits were dried in an oven (Conterm Poupinel; JP SELECTA S.A., Abrera, Barcelona, Spain) at 105 °C until a steady weight was achieved. Dry whole individual olive fruits were measured using an NMR Minispec NMS100 (Bruker Optik GmbH). Measurements were made in 30-mm-diameter glass tubes. The device was calibrated with 9 quantities of olive oil (from 0.05 g to 1.35 g), and a calibration line was built between NMR responses and corresponding oil weights. The oil content was given directly by software for each fruit in grams (OC_NMR_(g)). OCFW_NMR_(%) determined as a percentage on a fresh-weight basis by NMR for every fruit (*j*) belonging to the batch (*i*) was computed considering the FW(g) of each fruit according to (1).

1 $${OCF}{{W}}_{i,j{NMR}}( \% )=\frac{{O}{{C}}_{i{,}j{NMR}}(g)}{{F}{{W}}_{i{,}j}(g)}\cdot 100$$

In total, 278 dried olives from Subsamples 2 were stored in a dark and fresh place until the end of the harvest season. They were then moved to IRSTEA (Montpellier, France) facilities for spectral analysis. The relative reflectance hyperspectral images of each dry olive fruit were acquired using a vision system comprising a line-scan push broom camera (model HySpex SWIR-320m-e; Norsk Elektro Optikk, Skedsmokorse, Norway). The spectral range of the camera was 1000–2500 nm with spectral sampling every 6 nm. However, due to the low ratio signal-to-noise in the extreme of the spectra, only the range of 1003–2208 nm was considered (202 spectral bands). A halogen light source was used to illuminate the material to be imaged by the camera. The halogen bulb was switched on 30 min prior to taking a measurement to stabilize the light-source temperature drift and improve the spatial lighting uniformity. Reflectance images were obtained by scaling HSI images using a standard white. Absorbance images (−log_10_) were computed. The average spectrum of each fruit was considered for further analysis. Savitsky–Golay smoothing and differentiation algorithm were applied to the absorbance spectra: a polynomial of order three was fitted to a width of 21 wavelengths, and the first derivative function was applied to the smoothed absorbance spectra.

### Estimation models

To estimate the OCFW (%) and OC (g), partial least squares (PLS) regression was applied to the corresponding average spectra. The goodness of each estimation model was evaluated through the coefficient of determination (r^2^), standard error of calibration (SEC), standard error of cross validation (SECV), ratio of the prediction to deviation (RPD), number of latent variables (LV) and slope. All data analyses were performed using MATLAB_R2015a (The MathWorks, Natick, USA) and Statistica 13.3 (TIBCO Software Inc., California, USA) software.

**Figure 2:**
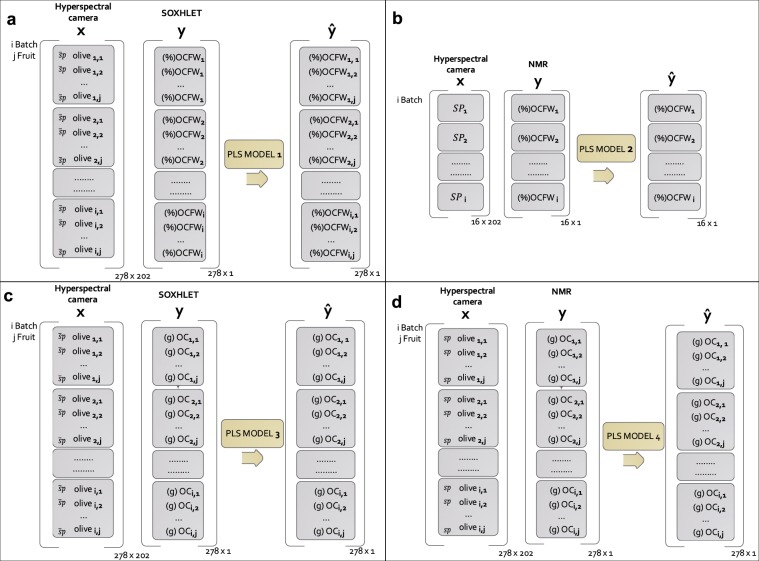
Schemes of the input and output of each PLS model. Input in MODEL 1 (**a**), MODEL 3 (**c**) and MODEL 4 (**d**) corresponds to 278 cases or olives belonging to 31 batches. Input for MODEL 2 (Eq. 4) corresponds to 16 cases or homogeneous batches of olives. MODEL 2 (**b**) is calibrated according to the variable determined by Eq. 3. MODEL 3 is calibrated according to the variable determined by Eq. 8.

In this work, two strategies were used to build models to estimate OCFW (%) (Fig. 2a,b):

- MODEL 1 to estimate OCFW_SOXHLET_ (%) in individual olives. The model used OCFW_SOXHLET_ (%) determined for each of the 31 batches as input in PLS regression. In this case, to match each average spectrum per olive with one reference data, OCFW_SOXHLET_ (%) was replicated by the number of olives of Subsample 2 of each batch. From estimations made by MODEL 1, it is possible to estimate the OC (g) on individual olives by (2).
  2 $${\widehat{OC}}_{i,jSOXHLET}(g)=\frac{{\widehat{OCFW}}_{i,jSOXHLET}( \% )}{100}\cdot F{W}_{i.j}(g)$$
- MODEL 2 to estimate OCFW_NMR_ (%) in a homogeneous batch. From MODEL 1 estimations of OCFW_SOXHLET_ (%), the 278 individual olives were re-clustered to build more homogeneous batches. Olives were sorted from OCFW_SOXHLET MODEL 1_ (%) of 14% to 32%, using steps of 1%. Sixteen homogeneous groups (all of them with a sufficient number of olives) were built. MODEL 2 used OCFW_NMR_ (%) determined for each one of the 16 batches as input in PLS regression. OCFW_NMR_ (%) in each new batch was calculated considering the OC_NMR_ (g) and FW (g) of the individual olives (*j*) belonging to the same batch (*i*) according to (3).
  3 $${OCF}{{W}}_{i{NMR}}( \% )=\frac{{\sum }_{j=1}^{n}{O}{{C}}_{{i},{j}{NMR}}(g)}{{\sum }_{j=1}^{n}F{W}_{i,j}(g)}\cdot 100,\,\,\,{n}={n}\,{of}\,{olives}\,{of}\,{the}\,{batch}$$
  To match each average spectrum per olive with one reference data per batch, the mean spectrum per batch was computed ((4).
  4 $${\overline{SP}}_{i}=\frac{{\sum }_{j=1}^{n}{\overline{sp}}_{i,j}}{n}$$
  Figure 2 shows that MODEL 1 is based on 278 cases or individual olives belonging to 31 “heterogeneous” batches, while MODEL 2 is based on 16 cases or “homogeneous” batches. The contribution of each olive (*j*) to the heterogeneity (*h*) of its batch (*i*) was computed according to (5), for the 31 “heterogeneous” batches (MODEL 1), and (6) for the 16 “homogeneous” batches (MODEL 2), equations adapted from Esbensena, K. H. *et al*.^21^.
  5 $${{h}}_{{i},{j}{het}}=\frac{{\widehat{OCFW}}_{i,jSOXHLET}( \% )-OCF{W}_{iSOXHLET}( \% )}{OCF{W}_{iSOXHLET}( \% )}\cdot \frac{F{W}_{i,j}}{F{W}_{l}}\,$$
  6 $${{h}}_{{i},{j}{\hom }}=\frac{{\widehat{OCFW}}_{i,jSOXHLET}( \% )-{\overline{\widehat{OCFW}}}_{iSOXHLET}( \% )}{{\overline{\widehat{OCFW}}}_{iSOXHLET}( \% )}\cdot \frac{F{W}_{i,j}}{{\overline{FW}}_{l}}\,$$
  where ${\widehat{OCFW}}_{i,jSOXHLET}( \% )$ is the OCFW (%) estimated by MODEL 1 for each olive (*j*) belonging to batch *i*, $OCF{W}_{iSOXHLET}( \% )$ is the OCFW(%) determined by Soxhlet in the reference laboratory for batch *i*, ${\overline{FW}}_{i}$ is the mean fresh weight of the olives belonging to batch *i*, and ${\overline{\widehat{OCFW}}}_{iSOXHLET}( \% )$ is the mean OCFW(%) estimated by MODEL 1 for batch *i* according to (7).
  7 $${\overline{\widehat{OCFW}}}_{iSOXHLET}( \% )=\frac{{\sum }_{j=1}^{n}{\widehat{OCFW}}_{i,jSOXHLET}( \% )}{n}$$
  On the other hand, two models were computed to estimate OC (g) in individual olives (Fig. 2c,d):
- MODEL 3 to estimate OC_SOXHLET_ (g) in individual olives. The model used OC_SOXHLET_ (g) determined for each fruit and calculated according to (8), as input in PLS regression.
  8 $$O{C}_{i,jSOXHLET}(g)=\frac{OCF{W}_{iSOXHLET}( \% )}{100}\cdot F{W}_{i,j}(g)$$
- MODEL 4 to estimate OC_NMR_ (g) in individual olives. The model used OC_NMR_ (g) directly determined by NMR for each fruit as input in PLS regression.

## Results and Discussion

### Reference analysis

**Figure 3:**
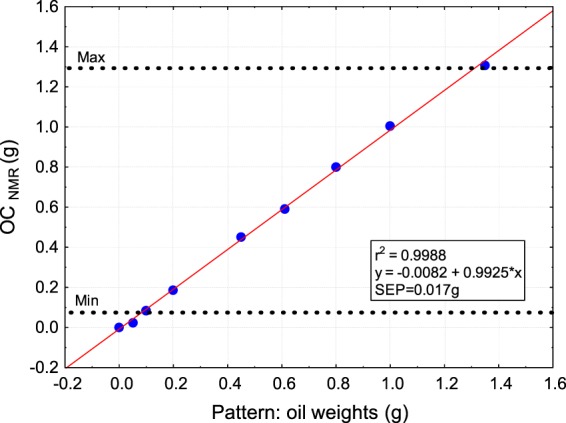
Calibration curve of the NMR instrument. The red line indicates the linear fit to the true values (blue dots) obtained by oil weights. The black dotted lines indicate the total range of OC measured in this population of olives.

The total range of OC_NMR_ (g) for individual olives varied from a minimum of 0.09 g up to a maximum of 1.29 g (n = 278), that is, all of olives were within the range of the calibration curve (Fig. 3). Such an OCNMR range is even wider than that reported by de la Rosa, *et al*.^9^ (0.1 g to 0.9 g in individual fruits).

Table 1 shows that the average olive FW was 2.07 g (±0.74) similar to the average FW per fruit reported by de la Rosa, *et al*.^9^ (2.13 g). The degree of heterogeneity of FW, in this olive fruit population, was high, with a CV of 35.88%. The average OC_NMR_ per fruit was 0.51 g (±0.24); de la Rosa, *et al*.^9^ reported an average OC per fruit of 0.49 g.

**Table 1:** Main statistics of the analytical parameters.

	Valid *n*	Mean	Minimum	Maximum	±Std. Dev.	CV (%)
FW (g)	278	2.07	0.77	4.51	0.74	35.88
OC_NMR_ (g)	278	0.51	0.09	1.29	0.25	49.60
OC_SOXHLET_ (g) by Eq. 8	278	0.51	0.16	1.33	0.24	46.61
OCFW_NMR_ (%) by Eq. 1	278	23.77	10.41	46.59	5.23	22.00
OCFW_SOXHLET_ (%)	31	23.96	18.92	31.54	3.48	14.54

Also in Table 1, OCFW (%) showed average values 23.77% (±5.23) and 23.96% (±3.48) as determined by NMR and Soxhlet respectively. The CV values being 22% for NMR and 14.54% for Soxhlet. Deblangey, *et al*.^18^ report a range of variation for OCFW_NMR_ from 17.1% to 35.5% which is much wider in our study: 10.41–46.59%.

**Figure 4:**
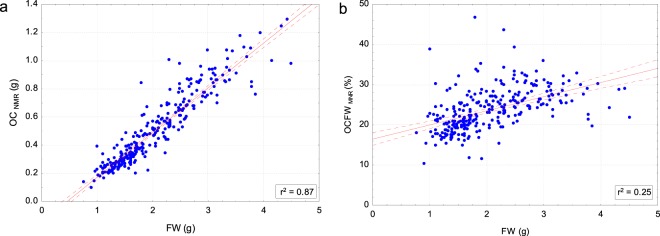
Scatterplots of FW (g) versus OC_NMR_ (g) at the left (**a**) and OCFW_NMR_ (%) determined by Eq. 1 at the right (**b**). The red line indicates the linear fit, and the dotted red lines indicate the confidence levels at 95%.

Figure 4(a) shows the correlation between FW (g) and OC_NMR_ (g). A strong uphill linear relationship is found r^2^ = 0.87 between these variables; even a stronger relationship (r^2^ = 0.94) was found by de la Rosa *et al*.^9^ in individual olives characterized for a breeding program. As expected, the quantity of oil (absolute value –g-) is higher for larger fruits, while OCFW_NMR_ refers to oil concentration.

Figure 4(b) shows the correlation between FW(g) and OCFW_NMR_(%) of individual olives. The determination coefficient (r^2^) is equal to 0.25, indicating that the relationship between the FW(g) of an individual and its OCFW(%) is moderate but non-relevant. The work of de la Rosa, *et al*.^9^ confirms this result with an r^2^ of 0.005 when comparing FW (g) of one fruit vs the OCFW (%) of its batch.

### Oil content referred to fresh weight in heterogeneous batches

**Figure 5:**
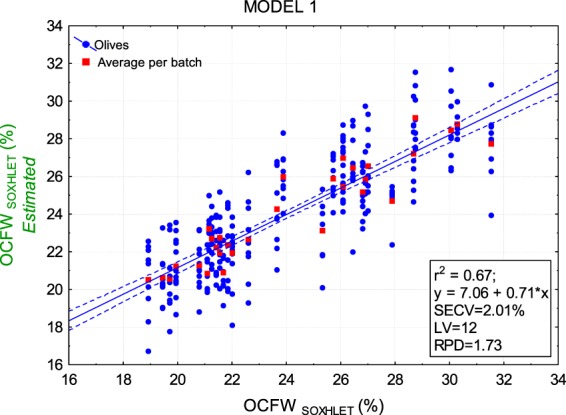
Scatterplot result of PLS MODEL 1. OCFW (%) measured using SOXHLET methods (x-axis) vs OCFW (%) estimated by MODEL 1 (y-axis). The blue line indicates the linear fit, and the dotted blue lines indicate the confidence levels at 95%.

Using spectral data, a first PLS model (MODEL 1) was built for estimating the oil concentration (OCFW (%)) since the OCFW (%) determined by batch is the most usual information used by industry to characterize the product previous to processing. The inputs for MODEL 1 covers the spectral information of each individual fruit plus one common reference per batch, OCFW_SOXHLET_ (%). Figure 5 shows the parameters that characterize the performance of MODEL 1. The coefficient of determination is low with r^2^ = 0.67, that is, the explained variance of the model is only 67% even when the number of LV is high (12). SECV is 2% and the RPD is between 1.5 and 2, indicating that the model can only segregate between high and low values^22^, which agrees with previous works.

Figure 5 shows the actual values of OCFW_SOXHLET_ (%) as compared to those estimated by MODEL 1. A vertical dispersion of data is found for the olives belonging to the same batch. The intra-batch SD estimated for OCFW_SOXHLET MODEL 1_ was 1.54% while the inter-batch SD reached 7.98%. Therefore, the intra-batch variability is 19.3% of the inter-batch variability, providing an idea of the heterogeneity of the estimated OCFW_SOXHLET MODEL 1_ per olive within each batch. A first question arises: is this variability due to an estimation error in MODEL 1 or to the intrinsic heterogeneity between the fruits included in the same batch? Assuming that the NMR technique is the best way to determine the OCFW (%) for individual olive fruits, the answer to this question could be found by comparing the estimations of MODEL 1 with the NMR values for individual fruits.

**Figure 6:**
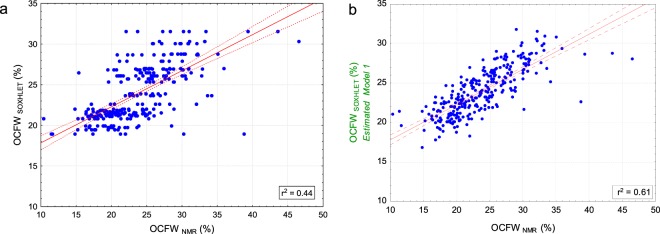
Scatter plots of OCFW_NMR_ (%) (Eq. 1) vs OCFW_SOXHLET_ (%) (**a**) and OCFW_SOXHLET_ predicted by MODEL 1 (**b**) for individual olives. The red line indicates the linear fit, and the dotted red lines indicate the confidence levels at 95%.

Figure 6(a) shows the OCFW_SOXHLET_ (%) per batch (31 values repeated to match 278) vs OCFW_NMR_ (%) per fruit (n = 278), pointing to a low relationship (r^2^ = 0.44) among them. An important difference was noted in the range of OCFW (%) determined by Soxhlet (18.9–31.5%) and that determined by NMR (10–47%), evidenced by the low value of the slope (0.44) of the fitted line (1 for the bisector). The intra-batch SD of OCFW_NMR_ was 3.76%, while the inter-batch SD was 11.6%, that is, the intra-batch variability of the OCFW_NMR_ was 32.4% of that inter-batch. Therefore the intra-batch variability is even higher when OCFW (%) is determined by NMR than when estimated by MODEL 1 using Soxhlet as reference analysis. This result is supported by the work of Deblangey, *et al*.^18^, which, under similar conditions, reported the higher sensitivity of the NMR methodology as compared to other reference analysis when determining OCFW (%) for individual fruits. Furthermore, Deblangey, *et al*.^18^ established that NMR generates the lowest precision errors.

Figure 6(b) shows OCFW_NMR_ (%) per fruit vs OCFW_SOXHLET MODEL 1_ (%) estimated per olive according MODEL 1; also in this case the batch effect is strongly attenuated (slope of 0.45). The correlation between the estimated values in MODEL 1 and true value was improved (r^2^ = 0.61), indicating that the estimations of MODEL 1 are nearer to the actual olive value of OCFW (%) for each olive as compared to that of the OCFW _SOXHLET_(%) determined per batch. Estimations with MODEL 1 expand the limits of OCFW to a range from 16% to 32%, though the model cannot properly estimate the OCFW (%) for individual olives beyond these limits, leading to saturated estimations especially at its upper limit (46.59%) according to OCFW_NMR_. Similar limits of OCFW (%) have been found by other researchers, with minimum and maximum OCFW values between 5% and 44%^8,11,19^.

**Figure 7:**
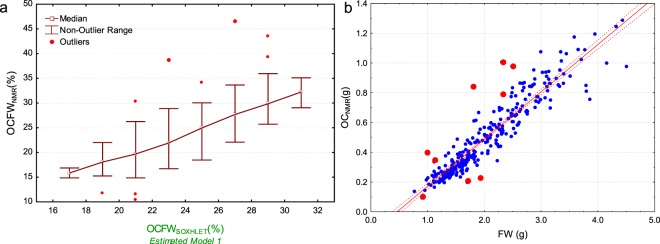
(**a**) Means with error plot using the integer mode to create grouping intervals, displaying outlier data. (**b**) Scatterplot of FW (g) vs OC_NMR_ (g). Red points indicate the location of outlier data.

Figure 7(a) identifies nine outliers (red circles) when comparing OCFW_SOXHLET MODEL 1_ vs OCFW_NMR_(%). In Fig. 7(b), the values of FW (g) vs OC_NMR_ (g) are plotted highlighting the outliers. The absolute values in grams of FW and OC determined for each of the highlighted data are within the range of calibration of NMR, as well as within the weight range for this population, however when combined in the computation of OCFW_NMR_ (%) lead to abnormal values, either being low or high. Considering that the manipulation of the sample is minimal (fresh whole fruit without pretreatment that is weighed in a balance with a scale accuracy of 0.001 g and then directly measured by NMR), it seems that these highlighted values are singular individuals detected by NMR and not measurements errors.

Thus, the high heterogeneity in the OCFW_NMR_ (%) of olives is demonstrated to be due to intrinsic differences among fruits even when they belong to the same batch, with MODEL 1 partially detecting such intrinsic differences.

When relating the OCFW_SOXHLET_ (%) of olives estimated by MODEL 1 with the actual value determined by NMR for each olive excluding the singular individuals a determination coefficient of r^2^ = 0.69 is obtained improving the r^2^ of 0.61 in Fig. 6(b). This fact corroborates the higher accuracy of OCFW (%) estimates with MODEL 1 for each olive with respect to OCFW_SOXHLET_.

### Oil content referred to fresh weight in homogeneous batches

**Figure 8:**
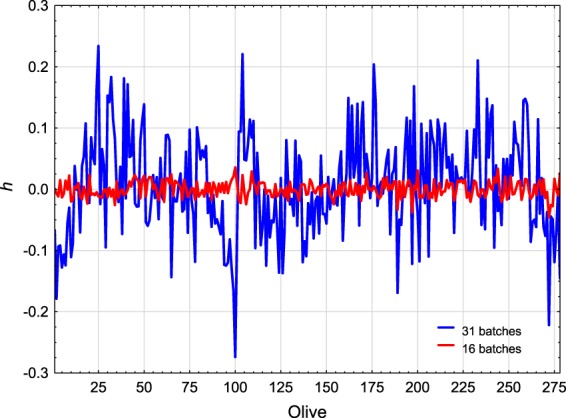
Contribution of each individual olive to the heterogeneity (*h*) of its batch. The blue line is *h* (Eq. 5) when 31 heterogeneous batches are considered, and the red line is *h* (Eq. 6) in the case of 16 homogeneous batches.

To generate homogeneous batches, the fruits were clustered into groups according to the values of OCFW_SOXHLET MODEL 1_ (%). Figure 8 plots the contribution of each olive to the heterogeneity (h) of its batch according to Eqs 5 and 6. The blue line points a high contribution of individuals to the heterogeneity of the batches when 31 cluster are considered. In the case of considering 16 groups, the heterogeneity (indicated by the red line) stays around 0, and thus selected as best option.

**Figure 9:**
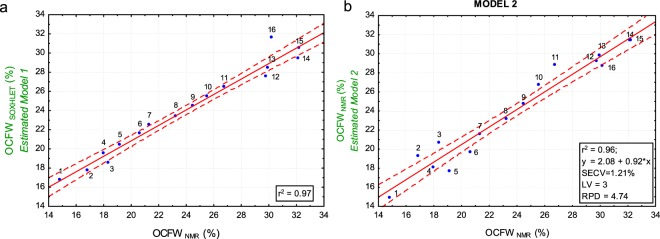
(**a**) Scatterplot of OCFW_NMR_ (%) per batch (Eq. 3) vs OCFW (%) per batch estimated by MODEL 1 (Eq. 7). (**b**) Scatterplot result of PLS MODEL 2. OCFW_NMR_ (%) per batch (Eq. 3) vs OCFW (%) estimated by MODEL 2 (y-axis) per batch. The red line indicates the linear fit, and the dotted red lines indicate the confidence levels at 95%. Point labels indicate de batch number.

Figure 9(a) shows the average estimate OCFW_SOXHLET_ (%) with MODEL 1 for each cluster (Eq. 7) compared to that of OCFW_NMR_ (%) (Eq. 3) achieving a high determination coefficient (r^2^ = 0.97). This means that 97% of the variance of OCFW_SOXHLET MODEL 1_ is explained by the actual OCFW (%) per cluster determined by NMR. Figure 9(b) shows the performance of MODEL 2, adjusted on the 16 homogenised spectra using OCFW_NMR_ as dependent variable. In this case the coefficient of determination was r^2^ = 0.96, with only 3 LV, indicating the high robustness of the model. SECV was 1.2%, RPD was 4.74, and the slope was 0.92, indicating that quantitative predictions are possible even at the extremes^22^.

**Figure 10:**
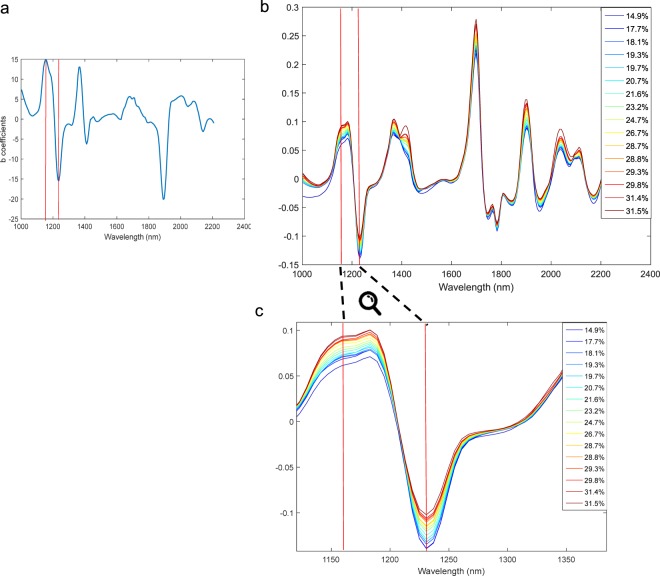
(**a**) *b* coefficients of PLS MODEL 2 where vertical red lines indicate peaks at 1153 and 1231 nm. (**b**) treated average absorbance spectra of 16 homogeneous groups plotted in the complete range and (**c**) it is shown the detail of these average spectra centred in the most informative wavelengths.

Figure 10(b) shows the average treated spectra of the 16 clusters considered as homogeneous, together with the loading of the wavelengths in MODEL 2 (Fig. 10a). Considering that 1200 nm is the main absorption band for fats and oils, a spectral zoom between the positive peak located at 1153 nm and negative peak located at 1231 nm is presented (Fig. 10c). It can be observed that the average spectra of each cluster are ordered according from lower to higher OCFW (%). This is not the case when considering the original batches (data not shown).

This approach proves that OCFW(%) estimates for individual fruits with MODEL 1 are accurate enough for classification purposes and can be used to generate homogeneous groups to reconfigure batches for a reference analysis, or either to select olives for breeding purposes.

### Calibration models to estimate the oil content (g)

**Figure 11:**
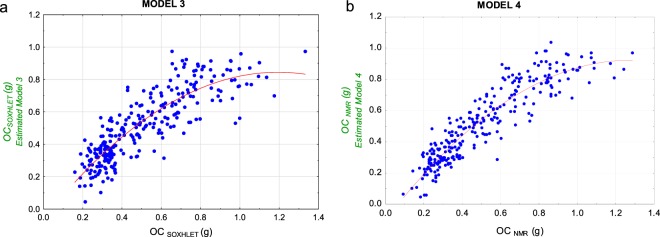
(**a**) Scatterplot result of PLS MODEL 3. OC_SOXHLET_ (g) (Eq. 8) vs OC (g) estimations per fruit (y-axis) (**b**) Scatterplot result of PLS MODEL 4. OC_NMR_ (g) vs OC (g) estimations per fruit (y-axis). The red line indicates the non-linear fit.

Figure 11 shows, from left to right, two models calibrated according to Eq. 8 with using OC_SOXHLET_ (g) (MODEL 3) and to OC_NMR_ (g) (MODEL 4) as dependent variables; both figures show a non-linear behaviour (banana-shaped distribution). High OC (g), usually corresponding to the largest fruits, are not accurately estimated with the PLS models and seem saturated above 1 gram of OC per fruit.

**Figure 12:**
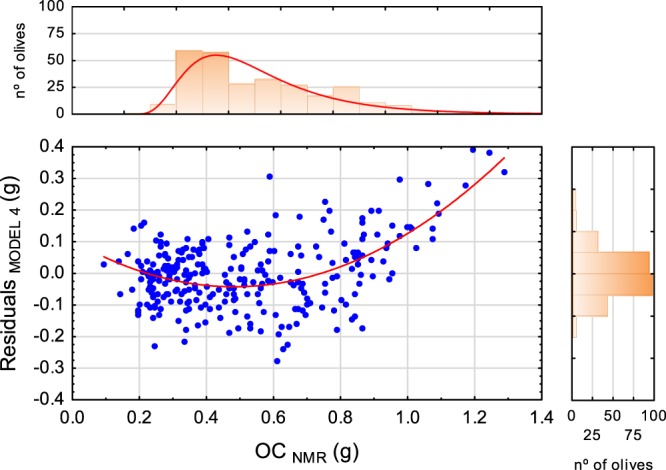
Scatterplot with marginal histograms of OC_NMR_(g) vs residuals of MODEL 4 (g). The red line indicates the non-linear fit.

Figure 12 confronts the analysis of the residuals in MODEL 4 by means of comparing the actual OC_NMR_ values (g) and the residuals of estimates. In this Figure the histograms of actual OC_NMR_ (g) and of estimate residuals are combined with corresponding scatterplot. Actual OC_NMR_ (g) does not follow a normal distribution; the distribution is positively skewed (skewness = 0.73) with a high occurrence for low OC (g). Thus, a heteroscedastic error may be inferred. Such lack of compliance with the restrictions for a linear regression can justify the low quality of the estimation with PLS models.

As stated by Beer´s law, which is valid only for transparent homogeneous materials, and the more practical approach of the Kubelka-Munk equation: f(C) = log(1/R), the information present in an NIR spectrum is related to the concentration of a given substance in a sample^23^. Most of the quantitative applications are targeted to determine major constituents in the sample, with usual detection limits of approximately 0.1% (m/m). The interactions of the light with the sample are limited to a restricted volume, implying that the change in the signal intensity is due to the major constituents that are inside this volume, representing the % in mass and not the total quantity of this constituents in the sample. Because spectroscopy is sensitive to concentration, in this case, to OCFW (%), but not to the total quantity of one compound (OC (g)), it could be considered a methodological error to calibrate a model directly with oil quantity in grams.

However, from a practical point of view, it is interesting to assess the mass of oil that is entering a mill. Therefore, it is necessary to answer whether it is possible to satisfy this requirement using NIR spectra. Comparing the OC_SOXHLET MODEL 1_ (g) (Eq. 2) with the true value determined per olive by NMR (OC_NMR_ (g)) were found a high determination coefficient (r^2^ = 0.93) and a low standard error of prediction (0.061 g). Therefore, when gathering the OCFW_SOXHLET MODEL 1_ (%) estimation and the FW (g) of each fruit in Eq. 2 the best estimation of the oil content in grams for individual olives is obtained.

Currently, vision machines are being developed to classify olives according to different quality parameters, such as colour and defects, previous to milling. These machines use multispectral vision cameras to extract the parameters included in the classification algorithms. In this state of development, it is perfectly possible to use the images to estimate the FW (g) of each olive^22^. As proven above, gathering the OCFW (%) information of the batch (i.e., by Soxhlet) together with the fresh weight (g) of olives would allow the estimation of the OC (g) per fruit, and thus the mas of oil (kg) which is entering the mill. Moreover, the use of multispectral cameras focused on the appropriate wavelengths, will lead to spectral models for OCFW (%) quantification that can be implemented in real-time, providing even a more accurate estimation.

## Conclusions

The complete seasonal heterogeneity in the OC of a commercial mill was characterized through systematic and representative sampling according to a factory standard (Soxhlet, %), together with the NMR oil actual value assessed on individual fruits, ranging from 10.4 to 45.6%, 0.09 to 1.29 g per olive.

The OC (g) estimated using a laboratory-top NMR instrument with specific calibration, is taken as the actual value in this study with a precision level of 0.017 g (0.8% of FW for an average fruit).

Current batch based assessment of the OC (Soxhlet, %) in mills only reproduces 44% of the underlying heterogeneity, despite being the factory standard used for payment to the farmer.

A PLS spectrometry model (1003–2208 nm) based on individual olives to estimate the OC reproduces 67% of batch variance and 60% of underlying heterogeneity. Therefore, spectrometry on individual olives helps to assess the variability of the oil content (%) in-mill even using batch values as the dependent variable.

It has been corroborated that it is a methodological error to develop PLS spectrometric models to directly estimate the OC (g) of the fruits since spectroscopy is sensitive to concentration but not to the total quantity of one compound (OC, g). However, the estimation of the OC (% fresh weight) by spectrometry on individual olives together with the assessment of fruit fresh weights (g) reproduces 93% of the variance of the oil content (g) in individual olives. A standard error of prediction of 0.061 g in the OC (g) (2.9% of FW for an average fruit) was reached through the combination of spectrometry and weight in individual olives, a value that approaches the potential limit according to the NMR reference (0.017 g) taken as the actual value.

The improvement in dealing with sample heterogeneity provided by the combination of spectrometry and olive fresh weights contributes to the fair rating of the product value, as well as to provide more accurate process settings in the mills.

It may be foreseen that developing olive grading lines combining spectrometric and physical properties of individual olives will become a commercial target in the near future for the olive oil industry.
